# Supplementary material for: Genome-Wide Identification and Co-Expression Networks of WOX Gene Family in Nelumbo nucifera
Source: Plants (Basel). 2024 Mar 4;13(5):720. doi: 10.3390/plants13050720 (PMC10933988; doi:10.3390/plants13050720)
Supplement: Supplementary file 1 [file plants-13-00720-s001.zip › Supplementary Figures.pptx]

## Slide 1
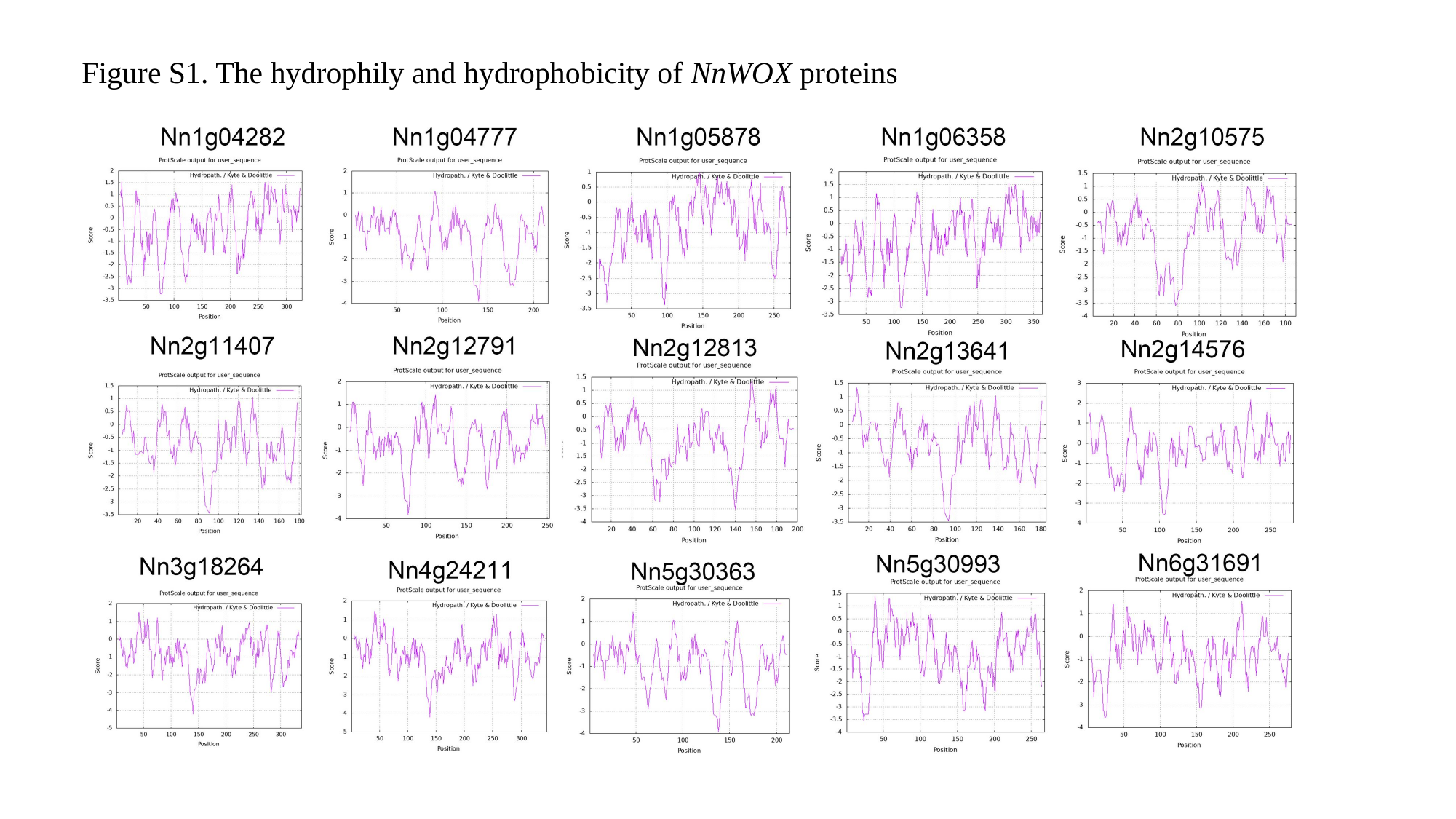

Figure S1. The hydrophily and hydrophobicity of NnWOX proteins

## Slide 2
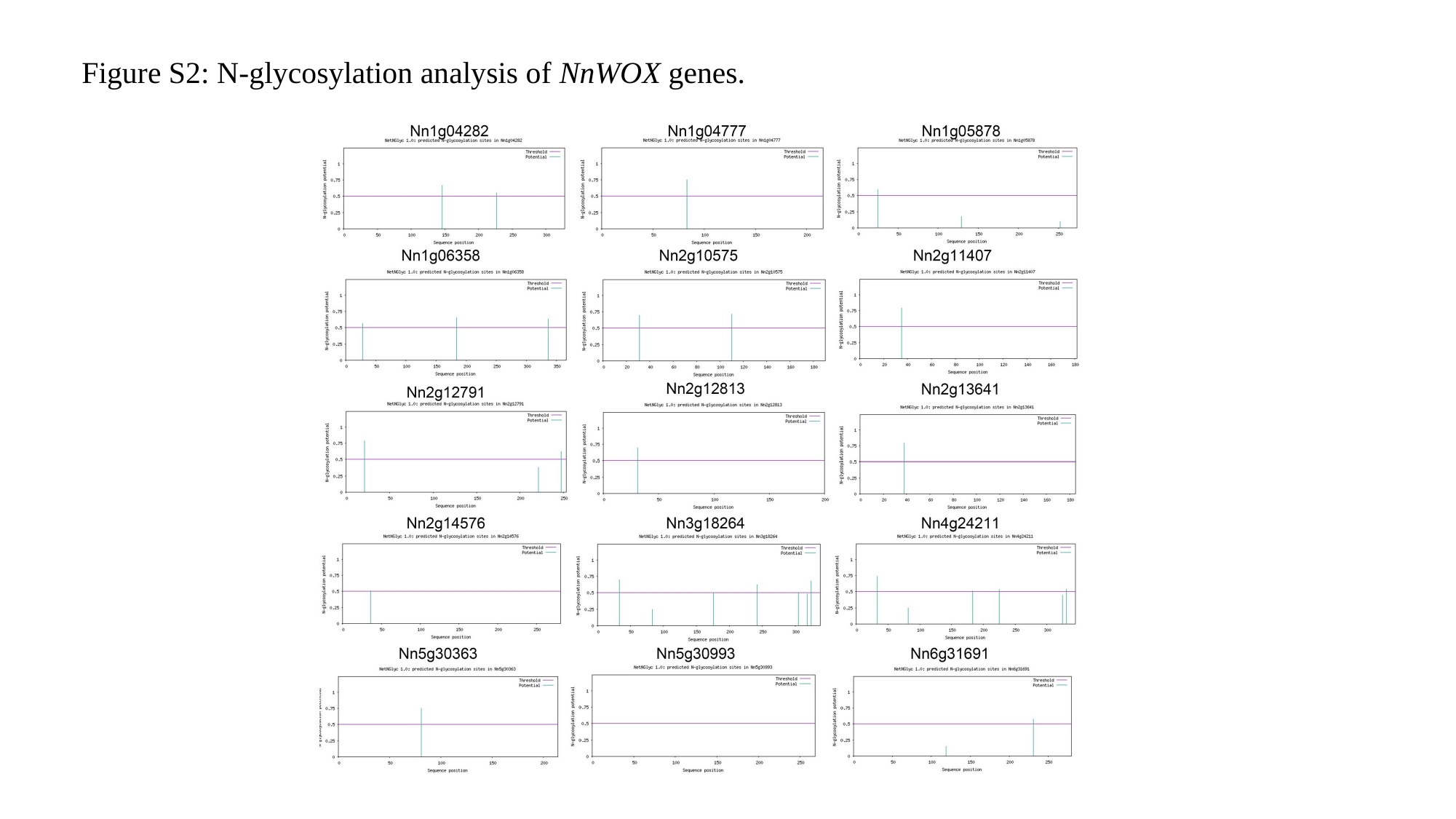

Figure S2: N-glycosylation analysis of NnWOX genes.

## Slide 3
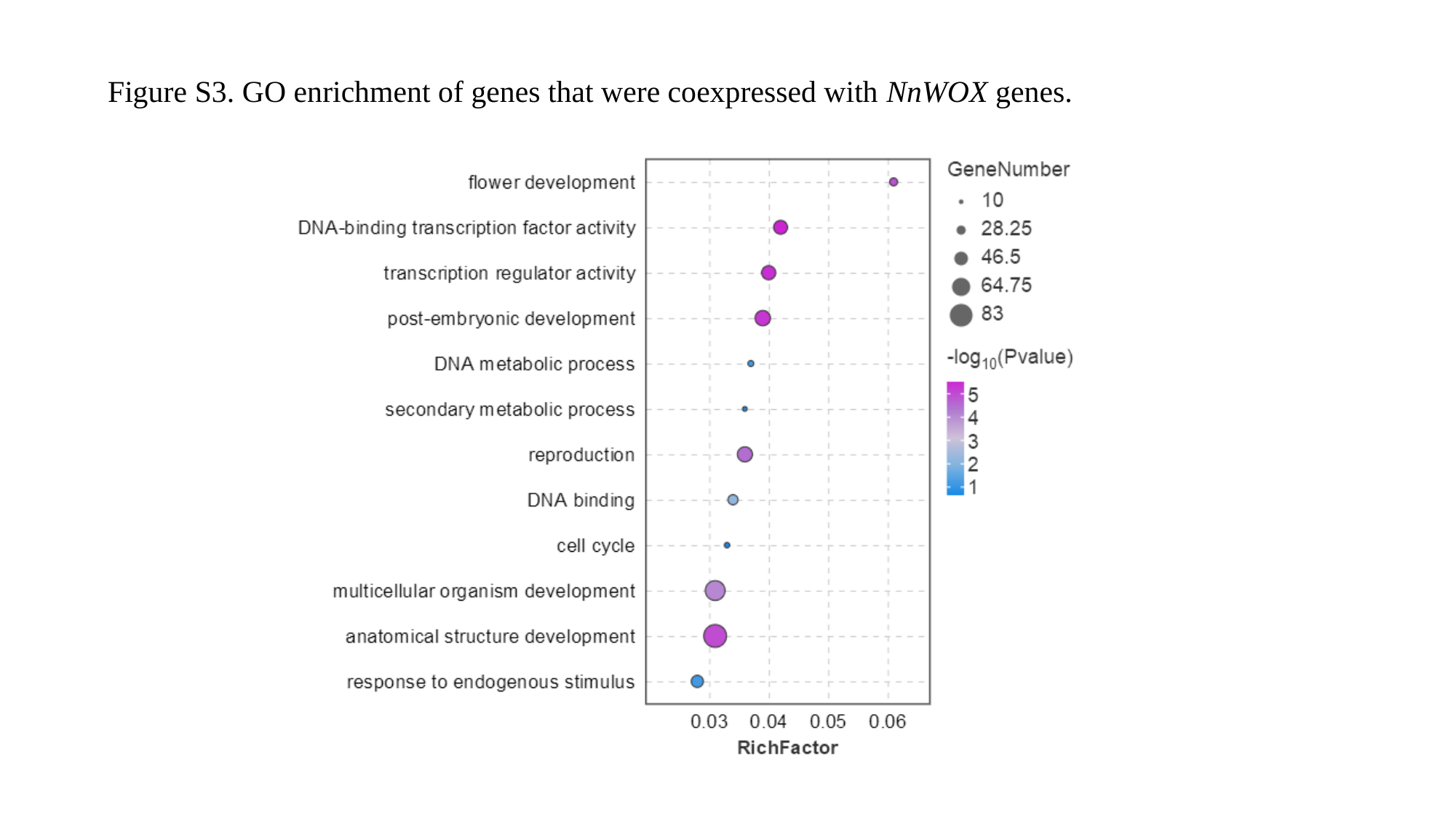

Figure S3. GO enrichment of genes that were coexpressed with NnWOX genes.
